# Lapatinib, a Dual EGFR and HER2 Tyrosine Kinase Inhibitor, Downregulates Thymidylate Synthase by Inhibiting the Nuclear Translocation of EGFR and HER2

**DOI:** 10.1371/journal.pone.0005933

**Published:** 2009-06-16

**Authors:** Hwang-Phill Kim, Young-Kwang Yoon, Jin-Won Kim, Sae-Won Han, Hyung-Seok Hur, Jinah Park, Ju-Hee Lee, Do-Youn Oh, Seock-Ah Im, Yung-Jue Bang, Tae-You Kim

**Affiliations:** 1 Cancer Research Institute, Seoul National University College of Medicine, Seoul, Korea; 2 Department of Internal Medicine, Seoul National University College of Medicine, Seoul, Korea; Bauer Research Foundation, United States of America

## Abstract

**Background:**

Epidermal growth factor receptor tyrosine kinase inhibitor (EGFR-TKI) has been shown to exert a synergistic antitumor effect when combined with fluoropyrimidine. This synergy may be attributable to the downregulation of thymidylate synthase (TS), which is frequently overexpressed in fluoropyrimidine-resistant cancer cells. However, the molecular mechanism underlying the downregulation of TS has yet to be clearly elucidated.

**Methodology and Principal Findings:**

In this study, we demonstrate that lapatinib, a dual TKI of EGFR and HER2 downregulates TS via inhibition of the nuclear translocation of EGFR and HER2. From our cDNA microarray experiments, we determined that a variety of nucleotide synthesis-related genes, including TS, were downregulated with lapatinib, and this was apparent in HER2-amplified cells. Targeted and pharmacologic inhibition assays confirmed that the dual inhibition of EGFR and HER2 is required for the more effective reduction of TS as compared to what was observed with gefitinib or trasutuzumab alone. Additionally, we determined that co-transfected EGFR and HER2 activate the TS gene promoter more profoundly than do either EGFR or HER2 alone. The translocation of EGFR and HER2 into the nucleus and the subsequent activation of the TS promoter were inhibited by lapatinib.

**Conclusions and Significance:**

These results demonstrate that lapatinib inhibits the nuclear translocation of EGFR and HER2 and downregulates TS, thus sensitizing cancer cells to fluoropyrimidine.

## Introduction

Lapatinib (GW572016, Tykerb) is a dual synthetic reversible inhibitor of EGFR and HER2 tyrosine kinases, and has been demonstrated to inhibit significantly the proliferation of cancer cells evidencing EGFR and/or HER2 overexpression both *in vitro* and *in vivo* [1]–[3]. At the intracellular level, lapatinib binds reversibly to the cytoplasmic ATP-binding site of the kinase, thereby preventing receptor phosphorylation [1], [4]. Lapatinib blocks ligand-activated signaling from multiple receptor combinations, including homo-and heterodimers of EGFR and HER2 [5]; preclinically, it inhibits the proliferation of trastuaumab-resistant cancer cells [2], [6]. Moreover, in contrast to trastuzumab, lapatinib can inhibit HER2 activation via ligand-induced heterodimerization or truncated HER2 receptors, and it has also proven effective in the treatment of PTEN-deficient breast cancer, thus illustrating the potential advantages of lapatinib over trastuzumab [7], [8].

Recently, lapatinib has been shown to exert beneficial effects in combination with capecitabine in patients with HER2-positive advanced breast cancer that has progressed after prior treatment with an anthracycline, a taxane, and trastuzumab [9]. In this trial, the time to progression of patients treated with lapatinib and capecitabine was prolonged significantly as compared to what was observed in patients treated solely with capecitabine (8.4 months vs. 4.4 months, p<0.001), which suggests that lapatinib may overcome trastuzumab resistance. However, another possible explanation for this observed synergistic effect would be that lapatinib may enhance sensitivity to capecitabine. In this regard, several lines of inquiry have demonstrated that EGFR-TKIs inhibited the expression of the transcription factor E2F-1, thereby inducing the downregulation of TS expression and activity, and mediating the synergistic interaction with 5-FU [10], [11]. However, the molecular mechanism underlying the downregulation of TS remains to be clearly elucidated. Fluoropyrimidines such as 5-FU are extensively utilized in the treatment of colorectal, breast, and aerodigestive tract cancers, and are intracellularly converted to 5-fluoro- deoxyuridine-monophosphate (FdUMP), thus forming a stable tertiary complex and inhibiting TS [12]–[14]. The results of several studies have demonstrated that the expression of TS functions as a key determinant of fluorpyrimidine sensitivity, and preclinical *in vitro* and *in vivo* studies have elucidated an inverse relationship between TS expression in cancer cells and fluoropyrimidine sensitivity [15]–[18]. Thus, EGFR TKI may represent a novel therapeutic strategy which can attenuate TS expression in cancer cells.

EGFR and HER2 are cell surface receptors which transduce mitogenic signals within the cells [19], [20]. However, the nuclear importation of EGFR and HER2 has been also demonstrated, although its biological significance remains unclear. EGFR has been detected in the nuclei of cancer cells and in primary tumor specimens of various origins, as well as in those of other highly proliferative tissues. While localized in the nucleus, EGFR may operate as a transcriptional regulator. It has been previously reported that nuclear EGFR regulates the expression of cyclin D1, inducible nitric oxide synthase (iNOS), and B-MYB genes via transactivational activity [21]–[23]. Furthermore, nuclear EGFR has been demonstrated to interact physically with signal transducer and activator of transcription 3 (Stat3) and E2F-1 [23], [24]. Aside from EGFR, other receptors in the EGFR family, including HER2, have also been detected within the nucleus [25], [26], but the biological significance of these receptors will require additional study.

In this study, we attempted to determine the manner in which lapatinib renders cancer cells susceptible to fluoropyrimidine. We determined that EGFR and HER2 existed within the nucleus, and that nuclear EGFR and HER2 bind to and activate the TS gene promoter. We further noted that lapatinib inhibits the nuclear translocation of EGFR and HER2, thereby induing a reduced association with the TS promoter. The lapatinib-mediated downregulation of TS was apparent in HER2-amplified cells; however, it was also noticeable in the wild-type cells. It is also important to note that the dual inhibition of EGFR and HER2 is the most effective method for achieving maximal TS downregulation. Taken together, these data show that lapatinib, a dual inhibitor of EGFR and HER2 TS, may prove useful not only as a targeted therapy, but also as a chemosensitizer of cytotoxic anticancer drugs in a specific subset of tumors.

## Results

### Lapatinib downregulates fluoropyrimidine-target genes including TS

**Figure 1:**
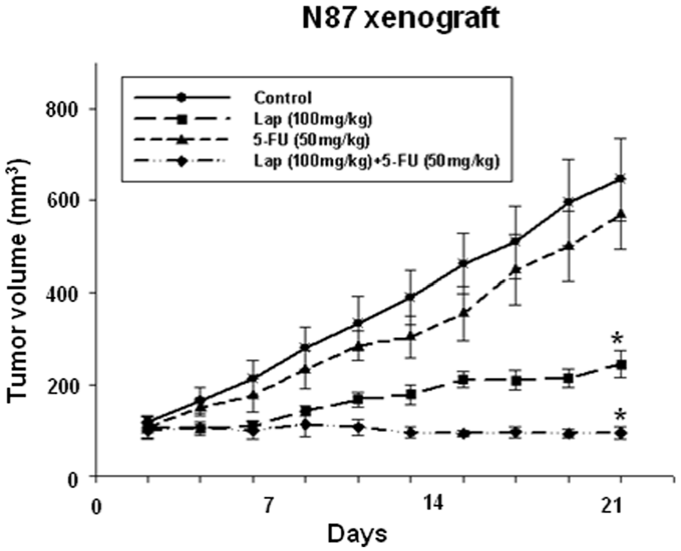
Combination of lapatinib and 5-FU potently inhibited tumor growth of N87-bearing xenografts. N87 cells (5×10^6^) were injected s.c. into nude mice with randomization (n = 6). Treatment with lapatinib (100 mg/kg, p.o., daily for 3 weeks) and 5-FU (50 mg/kg, i.p., once weekly for 3 weeks) was initiated once the tumors had achieved a volume of 50–100 mm^3^. Bars, SEM and repeated measures of ANOVA showed statistically significant effects (P<0.005) in the lapatinib and combination groups.

Recently, we reported that lapatinib evidences significant growth inhibitory activity in HER2-amplified gastric cancer (GC) cells and, in combination with 5-FU, results in a synergistic growth-inhibitory effect *in vitro* [27]. In the current study, these *in vitro* findings were confirmed in an *in vivo* context, where it was shown that lapatinib alone or a combination of lapatinib and 5-FU potently inhibited the tumor growth of HER2-amplified N87 GC cell-bearing xenografts (Fig. 1). These results further support the rationale for a cancer therapy based on a combination of lapatinib and fluoropyrimidine.

**Figure 2:**
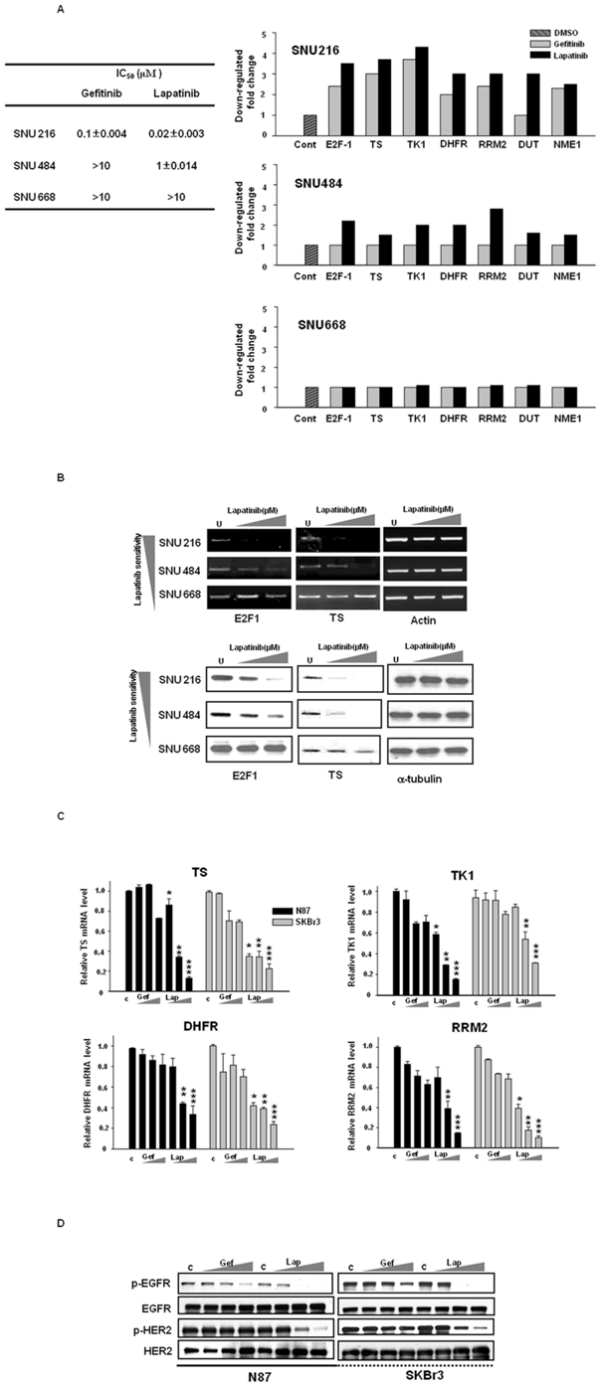
Lapatinib downregulates fluoropyrimidine-target genes. *A*, Downregulated fluoropyrimidine-sensitivity genes by oligonucleotide microarray based on analysis comparing DMSO/lapatinib-treatment versus DMSO/gefitinib-treatment samples at 24 hours of each treatment in SNU216, SNU484, or SNU668 cells. *B*, The indicated cells were treated with lapatinib (0.1, 1, 1 µmol/L) for 24 hours. mRNA levels of E2F1 and TS were assessed via RT-PCR (*upper*). β-Actin was utilized as a loading control. Western blots are provided for E2F1 and TS (*lower*). α-tubulin was employed as a loading control. *C and D,* N87, and SKBr3 cells were grown for 24 hours in the presence of gefitinib (0.01, 0.1, 1 µmol/L) or lapatinib (0.01, 0.1, 1 µmol/L). Western blots are shown for phosphorylated and total EGFR, and HER2. mRNA levels of TS, TK1, DHFR, and RRM2 were determined via quantitative real-time RT-PCR. Columns, means; *bars*, ±SD. *, P<0.05; **, P<0.05;***, P<0.05, gefitinib versus lapatinib at 0.01, 0.1, and 1 µmol/L doses. The expressed data are representative of three independent experiments.

It has been previously demonstrated that EGFR TKI, such as erlotinib/or gefitinib treatment with fluoropyrimidine, resulted in an synergistic inhibitory effect in non-small-cell lung cancer cells, possibly as the result of TS downregulation via the inhibition of EGFR signaling [10], [11]. Accordingly, we have speculated that lapatinib may be superior to EGFR TKI from the standpoint of chemosensitization to fluoropyrimidine. In order to address these issues and investigate the underlying molecular mechanisms, we first conducted a set of oligonucleotide microarray experiments to compare the effects of gefitinib with lapatinib from the perspective of the chemosensitization to fluoropyrimidine. We utilized three different GC cell lines, SNU216 (EGFR^WT^, HER2^Amp^), which is sensitive to lapatinib (IC_50_ 0.02 µM), or gefitinib (IC_50_ 0.1 µM), SNU484 (EGFR^WT^, HER2^WT^) which is moderately sensitive to lapatinib (IC_50_ m1 µM) and resistant to gefitnib (IC_50_>10 µM), or SNU668 (EGFR^WT^, HER2^WT^, K-ras^MT^) which is resistant to both (lapatinib IC_50_>10 µM, gefitnib IC_50_>10 µM; Fig. 2A, *left*). The expressions of representative nucleotide synthesis-related genes [E2F-1, TS, TK1 (thymidine kinase 1), DHFR(dihydrofolate reductase), RRM2 (ribonucleotide reductase M2 polypeptide), DUT (DUTP pyrophosphatase), NME1 (nuclear diphosphate kinase 1)] were reduced as the result of gefitinib or lapatinib treatment in TKI-sensitive cells. Fold reductions were decreased in proportion with drug sensitivity in these cases. For example, lapatinib downregulated gene expression in lapatinib-sensitive SNU216 and SNU484 cells, but did not affect gene expression in the lapatinib-resistant SNU668. As compared to gefitinib, lapatinib treatment induced higher fold reductions of all of the downregulated genes in SNU216 and SNU 484 cells (Fig. 2A, *right*). Thus, it is probable that the inhibition of both EGFR and HER2 appears to be more effective from the perspective of gene regulation. It is also crucial to note that lapatinib modestly downregulates gene expression in HER2 wild-type SNU484 cells, as well as in HER2-amplified cells. The transcription factor E2F-1 downregulates nucleotide synthesis-associated genes, including TS, and the change in E2F-1 as the result of lapatinib treatment was apparent in SNU216 and SNU484 cells, but was not noted in the SNU668 cells. All these data were confirmed via RT-PCR and Western blotting (Fig. 2B). Lapatinib downregulated TS mRNA and protein in a dose-dependent manner in the SNU216 and SNU484 cells, but not in the SNU668 cells (Fig. 2B). In HER2-amplified SNU216 cells that were sensitive to both, lapatinib suppressed gene expression more potently than gefitinib. These findings were confirmed in different HER2-amplified N87 and SKBr3 cells following treatment with either gefitinib or lapatinib. As is shown in Fig. 2C and Fig. S1, lapatinib consistently induced a profound reduction of TS, TK1, DHFR, or RRM2 mRNA in a dose-dependent manner as compared to gefitinib. Lapatinib effectively inactivated phosphorylated-EGFR and -HER2 at the same doses as used in Fig. 2C (Fig. 2D). Together, these results revealed that lapatinib downregulates a variety of nucleotide synthesis-related genes, including E2F-1 and TS. Moreover, the gene modulation effect of the dual inhibitor is superior to that of EGFR TKI, which is more apparent in HER2-amplified cells. Considering that these nucleotide synthesis-related genes are major determinants of fluoropyrimidine sensitivity, the dual inhibition of EGFR and HER2 TK by lapatinib appears to represent a promising strategy for the sensitization of cancer cells to fluoropyrimidines in a subset of tumors.

### Dual inhibition of EGFR and HER2 is required for TS downregulation

**Figure 3:**
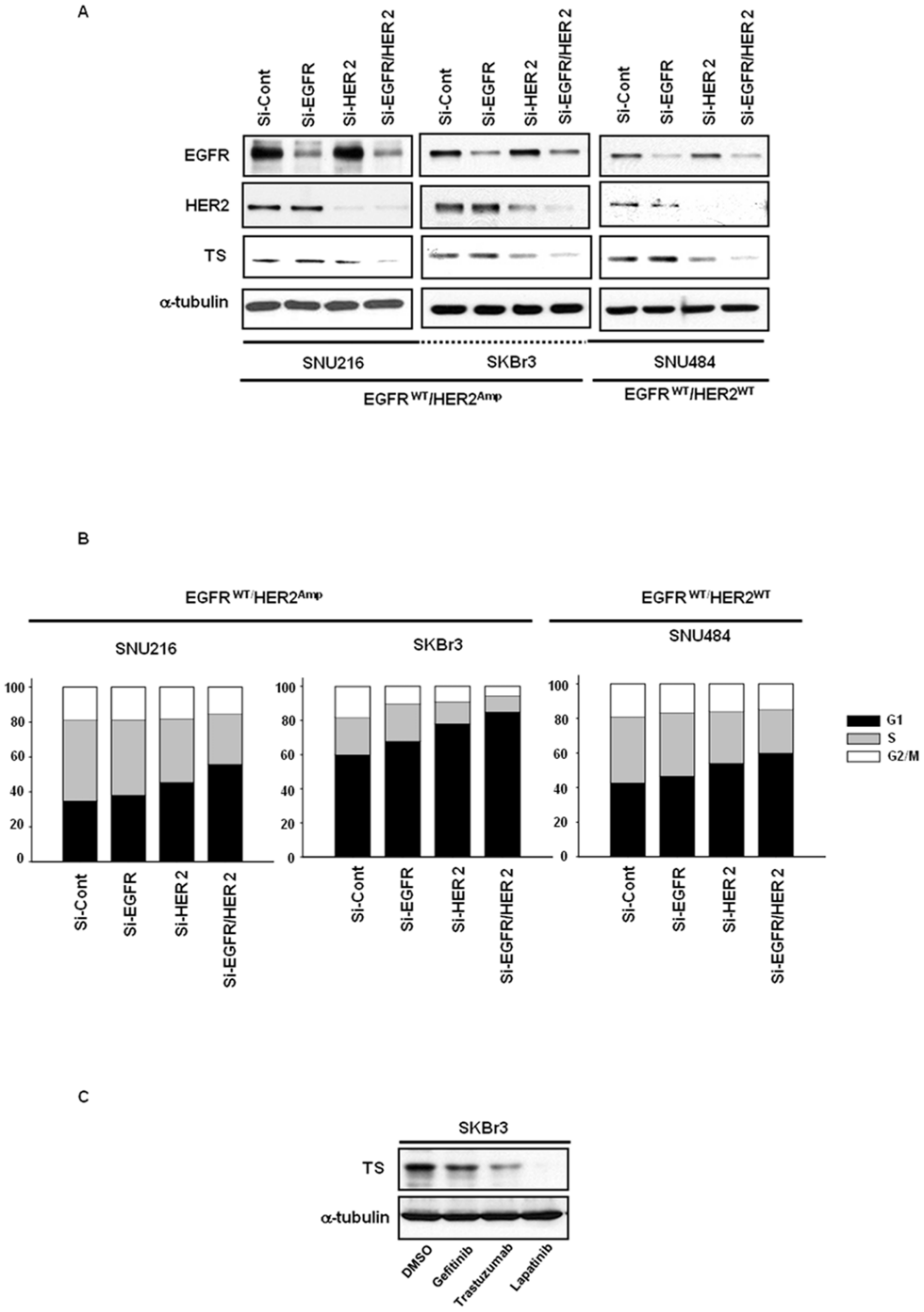
Dual inhibition of EGFR and HER2 kinase activity is required for TS downregulation. *A,* SiRNAs targeting endogenous EGFR, HER2, and both missense transcripts were transfected with SNU216, SKBr3, and SNU484 prior to 48 hours of lysate treatment. Whole cell extracts were Western-blotted with EGFR, HER2, and TS. α-tubulin was utilized as a loading control. *B*, The cells were fixed with 70% ethanol, stained with propidium iodide, and subjected to flow cytometric analysis. Proportions of cells in the G1, S, and G2-M phase were quantified using the ModFit LT program (Verity Software House Inc.); total percentages of G1, S, and G2-M phases are 100% in our data. *C,* SKBr3 cells were exposed for 24 h to gefitinib (1 µmol/L), trastuzumab (100 ng/ml), or lapatinib (1 µmol/L). Western blots are provided for TS. α-tubulin was used as a loading control. The expressed data are representative of three independent experiments.

In an effort to evaluate the biological and functional relevance of the dual inhibition of EGFR and HER2, we assessed TS protein levels following transfection with small interfering RNA (siRNA) oligonucleotides directed against EGFR, HER2, or both in HER2-amplified SNU216 and SKBr3, wild-type SNU484 cells (Fig. 3A). The dual inhibition of EGFR and HER2 effectively abolished TS expression in HER2-amplified SNU216, SKBr3, and N87 (data not shown), and similar effects were also observed in the HER2 wild-type cells. Consistent with the results observed with TS downregulation, co-transfection with EGFR and HER2 siRNA induced a profound G1-arrest of cancer cells (Fig. 3B). These results were confirmed by a pharmacological inhibitor experiment showing that lapatinib significantly downregulated the TS protein as compared with the effects of gefitinib or trastuzumab in SKBr3 cells (Fig. 3C). Thus, it can be concluded that the dual inhibition of EGFR and HER2 is required for the more effective downregulation of TS. Even in the HER2 wild-type cells, dual inhibition appears to be superior to single inhibition from the perspective of TS downregulation.

### Lapatinib inhibits the nuclear translocation of EGFR and HER2

**Figure 4:**
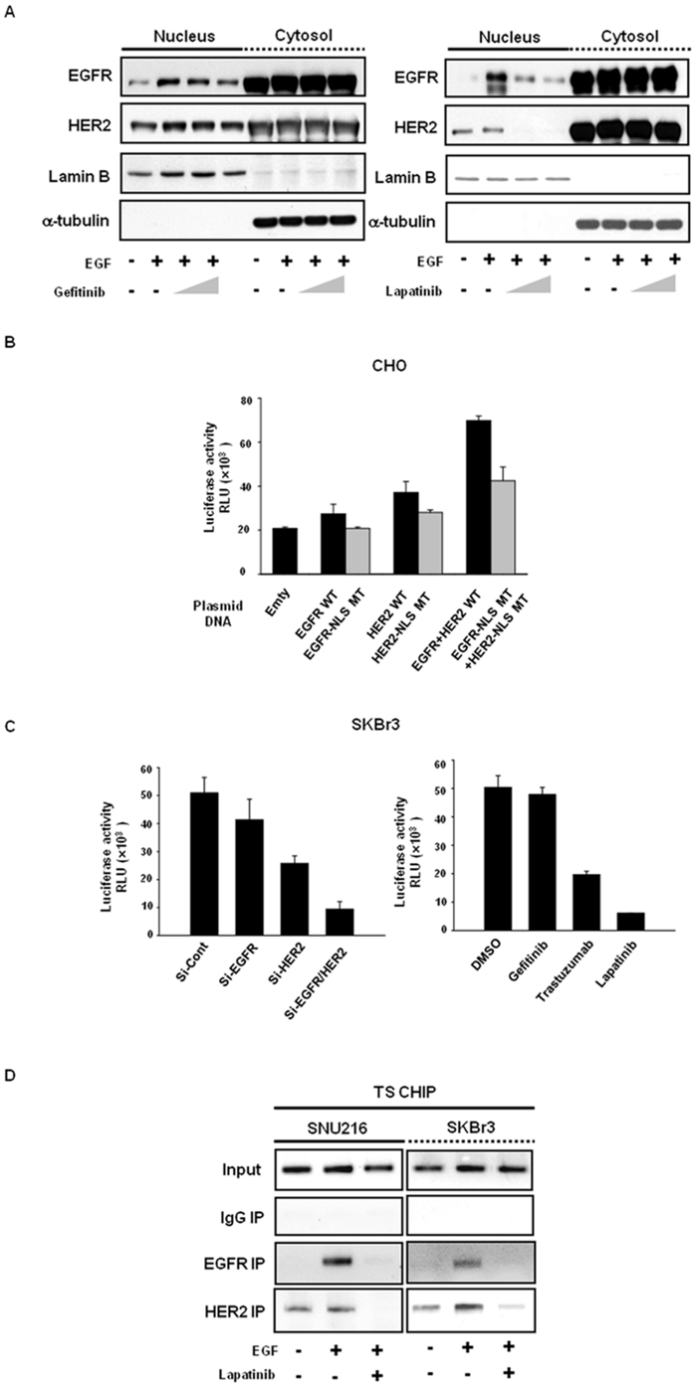
TS gene transcription by nuclear EGFR and HER2 is inhibited by lapatinib. *A,* Prior to harvesting, SNU216 cells were serum-starved for 24 hours, then grown for 3 h in the presence of gefitinib and lapatinib (0.1, 1 µmol/L), followed by 30 minutes of EGF (100 ng/mL) stimulation, then subjected to biochemical fractionation to separate the nucleus from the cytosolic material. Western blots are shown for EGFR and HER2. The loading controls were Laminin B (nucleus marker) and α-tubulin (cytosol marker). *B,* EGFR- and HER2-negative CHO cells were co-transfected with TS-promoter luciferase construct and plasmids encoding for EGFR-NLS WT/MT, HER2-NLS WT/MT, or both, in addition to mock plasmid. Following 24 hours of serum-starvation, the cells were stimulated for 6 hours with EGF and subjected to luciferase assays. *C*, TS-promoter luciferase construct and Si-RNAs targeting endogenous EGFR, HER2, and both missense transcripts were transfected with SKBr3, prior to 48 hours of lysate treatment (*left*). The cells transfected with TS-promoter luciferase construct were pretreated with gefitinib (1 µmol/L), trastuzumab (100 ng/ml), or lapatinib (1 µmol/L) for 24 hours (*right*). Luciferase activity was then determined. Columns, means; *bars*, ±SD. *D,* SNU216 and SKBr3 cells were serum-starved for 24 hours, then grown in the presence of lapatinib (1 µmol/L) for 3 hours, followed by 30 minutes of EGF (100 ng/mL) stimulation, and then subjected to CHIP analysis. Immunoprecipitation was then conducted with anti-EGFR, anti-HER2, or normal rabbit IgG. The TS promoter region which contained essential promoter regions and E2F-1 binding sites was PCR-amplified. Input nuclear DNA were utilized as a PCR control. The expressed data are representative of three independent experiments.

While located in the nucleus, EGFR or HER2 has been shown to function as a transcription factor for DNA repair and synthesis genes [21]. In order to determine whether gefitinib or lapatinib influences the nuclear localization of EGFR and HER2, we assessed the cytoplasmic-to-nuclear distribution of EGFR and HER2 following EGF stimulation. In HER2-amplified SNU 216, EGFR was translocated into the nucleus with EGF-stimulation, and HER2 was stably detected both in the cytosol and nucleus. After lapatinib treatment, both nuclear EGFR and HER2 were significantly reduced in these cells, whereas gefitinib treatment induced modest reduction of nuclear EGFR. In contrast, cytosolic EGFR and HER2 were not altered by either gefitinib or lapatinib (Fig. 4A). In the case of EGFR, lapatinib appeared to inhibit the ligand-dependent translocation of EGFR into the nucleus. However, as HER2 has already been detected in the absence of ligand in HER2-amplified cells, lapatinib may inhibit preexisting translocated nuclear HER2, independently of the ligand. Although its molecular mechanism will require further investigation, lapatinib appears to reduce levels of nuclear EGFR and HER2, possibly via the inhibition of ligand-dependent or independent translocation of EGFR and HER2.

### Interaction of nuclear EGFR and HER2 with TS promoter is inhibited by lapatinib

We subsequently assessed the effects of nuclear EGFR and HER2 on TS gene transcription. First, we compared TS promoter activity according to the transfection of EGFR and HER2 nuclear localization signal (NLS) WT or MT in both EGFR and HER2-negative CHO cells. Following transient transfection with EGFR-NLS WT, HER2-NLS WT or both, we noted that TS promoter activity was increased to a greater degree by forced HER2 expression than by forced EGFR expression, and was activated more profoundly by dual-expression than by single-expression. By way of contrast, after transfection with EGFR-NLS MT, HER2-NLS MT, or both, TS promoter activation was lower than the effects of WT (Fig. 4B). Consistently, the knockdown of EGFR and/or HER2 and lapatinib treatment significantly reduced the ability of the TS promoter in response to EGF in SKBr3 cells, as compared to what was observed with gefitinib or trastuzumab alone (Fig. 4C). CHIP demonstrated that EGF-induced nuclear EGFR and HER2 bind to the TS promoter in SNU216 and SKBr3 cells, and that their association was abolished by lapatinib (Fig. 4D). Collectively, these results reveal that nuclear EGFR and HER2 activate TS gene transcription via binding to the TS promoter, and that the TS promoter-bound EGFR and HER2 are inhibited by lapatinib, ultimately resulting in the downregulation of TS.

## Discussion

In this study, we have demonstrated that lapatinib, a dual inhibitor of EGFR and HER2 TK, effectively downregulates a variety of nucleotide synthesis-related genes, including TS, and exhibits activity superior to that of gefitinib not only in HER2-amplified cells, but also in wild-type cells. As a mechanism, we have determined, for the first time, that nuclear EGFR and HER2 activate TS gene transcription, and that EGFR and HER2-bound TS promoter activities are inhibited by lapatinib treatment.

Traditionally, EGFR and HER2, once activated, form a homo- or heterodimer and transduce the mitogenic signal into the nucleus [21]. However, it has also been demonstrated that ligand-activated EGFR or overexpressed HER2 can form a homo- or heterodimer, and that these are also translocated into the nucleus. Therefore, nuclear EGFR and/or HER2 can operate as transcription factors, activating a variety of genes, including iNOS, B-MYB, and COX-2 [21]–[25]. Considering that nuclear EGFR and HER2 can function as transcription factors, it can be plausibly surmised that other genes involved in cell proliferation might also be regulated by nuclear EGFR and HER2. In the current study, we demonstrated that TS is activated by nuclear EGFR and HER2. In the context of TS activation, it appears that EGFR and HER2 may perform a pivotal role, as the dual inhibitor, lapatinib, or the double knockdown of EGFR and HER2, evidenced the maximal TS inhibitory effects. It is also worth noting that lapatinib is superior to gefitinib with regard to gene regulation, which also emphasizing the significance of the dual inhibition of EGFR and HER2.

In SNU216 cells evidencing HER2 overexpression, we determined that nuclear EGFR and HER2 are induced in a ligand-dependent and -independent fasion, respectively. The nuclear translocation of EGFR upon ligand stimulation is noticeable in these cells and is inhibited by lapatinib, which implies that the inhibition of EGFR is also required in HER2-amplified cells. It is also conceivable that nuclear EGFR and HER2 may form a homo- and heterodimer, which would bind to the promoters of target genes such as TS. We have previously determined that lapatinib inhibits the heterodimer of EGFR and HER2 in cancer cells [5]. Because lapatinib reduced the levels of nuclear EGFR and HER2, we speculated that lapatinib might also destabilize the levels of hetero- or homodimers of EGFR and HER2 in the nucleus. In HER2 wild-type cells, lapatinib exerted modest gene downregulation effects, as is shown in Figs. 2A and 2B. Therefore, it appears that lapatinib can be tested for the purpose of gene modulation, regardless of the HER2 status.

Collectively, the data provided in this study demonstrate the molecular mechanisms underlying the synergy between lapatinib and fluoropyrimidine. In the future, lapatinib should be investigated as a chemosensitizing agent that enhances cytotoxicity or circumvents resistance against anticancer drugs, not only in HER2-amplified, but also in HER2 wild-type tumors.

## Materials and Methods

### Reagents

Gefitinib was kindly provided by AstraZeneca, and lapatinib was generously provided by GlaxoSmithKline. Trastuzumab was kindly provided by Roche. 5-FU was obtained from Choong Woe (Seoul, Korea). Epidermal growth factor (EGF) was purchased from Sigma-Aldrich (St. Louis, MO).

### Cell Culture

Four human gastric cancer cells (SNU216, SNU484, SNU668, N87), human breast cancer cells (SKBr3), and Chinese hamster ovary cells were grown at 37°Cunder 5% CO_2_ in RPMI-1640 or DMED culture media containing 10% fetal bovine serum (WELGENE Inc., Korea). The mycoplasma free cells were purchased from the Korean Cell Line Bank (Seoul, Korea, ref[28]) or the American Type Culture Collection.

### N87 Xenografts

All animal experiments were approved by the Institutie Laboratory Animal Resources Seoul National University and Use Committee. Six-to-eight-week-old female BALB/c athymic (nu+/nu+) mice were purchased from Central Lab Animal Inc. (Seoul, Korea). The initial body weight of the animals at the time of arrival was between 18 and 20 g. Mice were allowed to acclimatize to local conditions for 1 week before being injected with cancer cells. Tumors were induced by injecting H1975 cells (5×10^6^) subcutaneously into the right flank of mice. The tumors were then measured twice a week using calipers, and the tumor volume in mm^3^ was calculated according to following formula: {(width)^2^×(height))/2}. When tumors had reached a volume of 50–100 mm^3^, treatment with either lapatinib, 5-FU, a combination of lapatinib and 5-FU, or a vehicle control was initiated. Lapatinib were administered via oral gavage at a concentration of 100 mg/kg in 0.5% (W/W) hydroxypropylmethylcellulose (HPMC) with 0.1% (W/W) Tween80 (Sigma) in sterile milli-Q water Monday through Friday for 3 weeks. A dose of 50 mg/kg of 5-FU was given intraperitoneally once weekly for 3 weeks. Statistical analysis to compare tumor sizes in xenograft-bearing mice was performed with ANOVA. Differences between groups were considered statistically significant if *P*<0.05.

### cDNA Microarray

SNU216, SNU484, and SNU668 cells were grown for 24 hours in the presence of gefitinib (1 µmol/L) or lapatinib (1 µmol/L), after which they were lysed. The total RNA was then processed and hybridized to an Affymetrix Genechip HG-U133 set (Affymetrix, Santa Clara, CA) via a DNA link (Seoul, Korea) according to the manufacturer's protocols. All samples were analyzed and reported according to MIAME guidelines. The GeneExpress Software System Fold Change Analysis tool was used to identify all present genes expressed at least 2-fold greater in the drug-treated cells compared with DMSO-treated cells. For each gene fragment, the ratio of the geometric means of the expression intensities in DMSO treated cells and the drug treated cells cells was calculated, and the fold change was then calculated on a per fragment basis. Confidence limits were calculated using a two-sided Welch modified *t* test on the difference of the means of the logs of the intensities.

### Reverse Transcription-PCR and Real-Time PCR Analysis

Specific mRNAs were semiquantitated via reverse transcription (RT-PCR) or real-time PCR with the iCycler IQ detection system (Bio-Rad Laboratories, Hercules, CA) using SYBR green I (Molecular Probe, Eugene, OR) in triplicate reactions. The primers used in the PCR reaction were as follow: E2F1, forward primer5′- ACGCTATGAAACCTCACTAAA-3′ and reverse primer5′-AGGACATTGGTGATGTCATA-3′, TS, forward primer 5′- TCTGGAAGGGTGTTTTGGA-3′ and reverse primer 5′-CCTCCACTGGAAGCCATAAA-3′, TK1, forward primer 5′- CAGCTTCTGCACACATGAC-3′ and reverse primer 5′-AGTGCAGCCACAATTACGG-3′, DHFR, forward primer 5′- TCCATTCCTGAGAAGAATCGACCTT-3′ and reverse primer 5′-CACAAATAGTTTAAGATGGCCTGGG-3′, RRM2, forward primer 5′-GTGGAGCGATTTAGCCAAGA-3′ and reverse primer 5′-TGACCTCTTTGTCCCCAATC-3′, DUT, forward primer 5′- CCCTTCTGGGTGTTATGGGAAGA-3′ and reverse primer 5′-CCAGCTCCTACATCAATAAAGTGTTT-3′, NME1, forward primer 5′- TTCACCCTGAGGAACTGGTAGATT-3′ and reverse primer 5′-GTGGTCTGCCCTCCTGTCA-3′, ACTIN, forward primer 5′- AGAGCTACGAGCTGCCTGAC and reverse primer 5′- GGATGCCACAGGACTCCA-3′.

### Antibodies and Western Blotting

Antibodies against E2F-1, α-tubulin, and Lamin B were purchased from Santa Cruz Biotechnology (Santa Cruz, CA). p-EGFR (pY1068), EGFR, p-HER2 (pY1221/1222), HER2, and TS antibody was acquired from Cell Signaling Technology (Beverley, MA), or NeoMarkers (Fremont, CA). Cultured cells that had reached ∼70% to 80% confluence were used for protein analyses. The cells were treatmented with different conditions as described. The cells were lysed in RIPA buffer on ice for 15 min (50 mmol/L Tris-HCl pH 7.5, 1% NP-40, 0.1% Na deoxycholate, 150 mmol/L NaCl, 50 mmol/L NaF, 1 mmol/L sodium pyrophosphate, 1 mmol/L sodium vanadate, 1 mmol/L nitrophenolphosphate, 1 mmol/L benzamidine, 0.1 mmol/L PMSF, 0.1 mmol/L aprotinin, 0.1 mmol/L leupeptin, 0.1 mmol/L pepstatin A) and centrifuged at 13,000 rpm for 20 min. Samples containing equal amount of total protein were resolved in SDS-polyacrylamide denaturing gel, transferred to nitrocellulose membranes, and probed with antibodies. Detection was performed using an enhanced chemiluminescence system (Amersham Pharmacia Biotech).

### Cell Cycle Analysis

Cells were washed twice in phosphate buffered solution (PBS), fixed in 70% ethanol, and stored at −20°C until required for analysis. Before analysis, cell suspensions were washed with PBS, and digested with RNase A (50 µg/ml) for 15 minutes at 37°C and then stained with propidium iodide (50 µg/ml). Cell DNA contents (10,000 cells/experimental group) were determined using a FACSCalibur flow cytometer (Becton Dickinson Biosciences, San Jose, CA) equipped with a ModFit LT program (Verity Software House Inc.), as previously described [29].

### Nuclear Fractionation

SNU216, SKBr3, and N87 were serum-starved for 24 h and stimulated with EGF (100 ng/ml) for 30 min and then collected for lysis. Nuclear fractions were prepared using NE-PER extraction reagents (Pierce Chemical, Rockford, IL), according to the manufacturer's instructions. Briefly, after removal of the cytoplasmic fraction using cytoplasmic extraction reagents, the insoluble pellet obtained was resuspended in nuclear extraction reagent (100 mmol/L KCl, 10 mmol/L HEPES, pH 7.9, 10% glycerol, 1 mmol/L dithiothreitol, 5 mmol/L MgCl_2_, 0.1% NP-40, and 10 mmol/L NaF) containing protease inhibitors. After vigorously vortexing every 10 min during incubation on ice for 40 min, the nuclear fraction was isolated by centrifugation. Westernblotting for Lamin B and α-tubulin was performed to confirm the nuclear fraction and to exclude cytoplasmic contamination, respectively.

### EGFR and HER2-NLS Mutant Constructs

EGFR-NLS MT (645-47, RRR>AAA) and HER2-NLS MT (667-68, RR>GG) were induced in the cDNA using a QuickChangeTM Site-directed mutagenesis kit (Stratagene, La Jolla, CA), according to the manufacturer's protocol, as previously described [30], [31].

### Luciferase Reporter Assay

After cotransfection with pGL3-TS-Luc and EGFR or HER2 plasmids, the cells were lysed and the TS-luciferase activity was evaluated using a TR717 microplate luminometer (Applied Biosystems, Foster City, CA), in accordance with the manufacturer's instructions. The human TS promoter region was designed as previously described [32]. pCMV-β-Gal was also transfected in order to normalize the transfection efficiencies.

### Chromatin Immunoprecipitation Assay (CHIP)

SNU216 and SKBr3 were serum-starved for 24 h and stimulated with EGF (100 ng/ml) for 30 min. Briefly, cells were cross-linked by addition of 1% formaldehyde for 10 min and glycine was added (125 mmol/L final) for 5 min to stop the cross-linking reaction. Cells were then lysed with a lysis buffer and sonicated. One-tenth of the total chromatin lysate was used for purification of total genomic DNA. The rest of the lysate was used for immunoprecipitation with EGFR or HER2 antibody. After the collection of immunoprecipitates using protein G agarose, protein-DNA complexes were eluted and heated at 65°C to reverse cross-linking. After digesting proteins by proteinase K, DNA fragments were purified using the QIAquick PCR purification kit (QIAGEN, Valencia, CA). Either total or immunoprecipitated DNA were analyzed by PCR of 30 or 35 cycles, respectively, at 94°C for 30 s, 56°C for 30 s, and 72°C for 1 min. Specific sequences of the TS promoter in the immunoprecipitates were detected by PCR with primer: forward, 5′- TGGCGCACGCTCTCTAGAGC-3′ and reverse, 5′- GACGGAGGCAGGCCAAGTG-3′. SNU216 and SKBr3 were serum-starved for 24 h and stimulated for 30 min with EGF (100 ng/ml). The primer utilized for the CHIP assay harbors TS essential promoter regions and E2F-1 binding sites [22], [33].

### Statistics

The statistical significance of the results was calculated by unpaired Student's t test, and P values of <0.05 were considered to be statistically significant.

## Supporting Information

Figure S1 (0.22 MB PDF)
