# Supplementary figures and images for: Lapatinib, a Dual EGFR and HER2 Tyrosine Kinase Inhibitor, Downregulates Thymidylate Synthase by Inhibiting the Nuclear Translocation of EGFR and HER2
Source: PLoS One. 2009 Jun 16;4(6):e5933. doi: 10.1371/journal.pone.0005933 (PMC2691960; doi:10.1371/journal.pone.0005933)

Figure S1

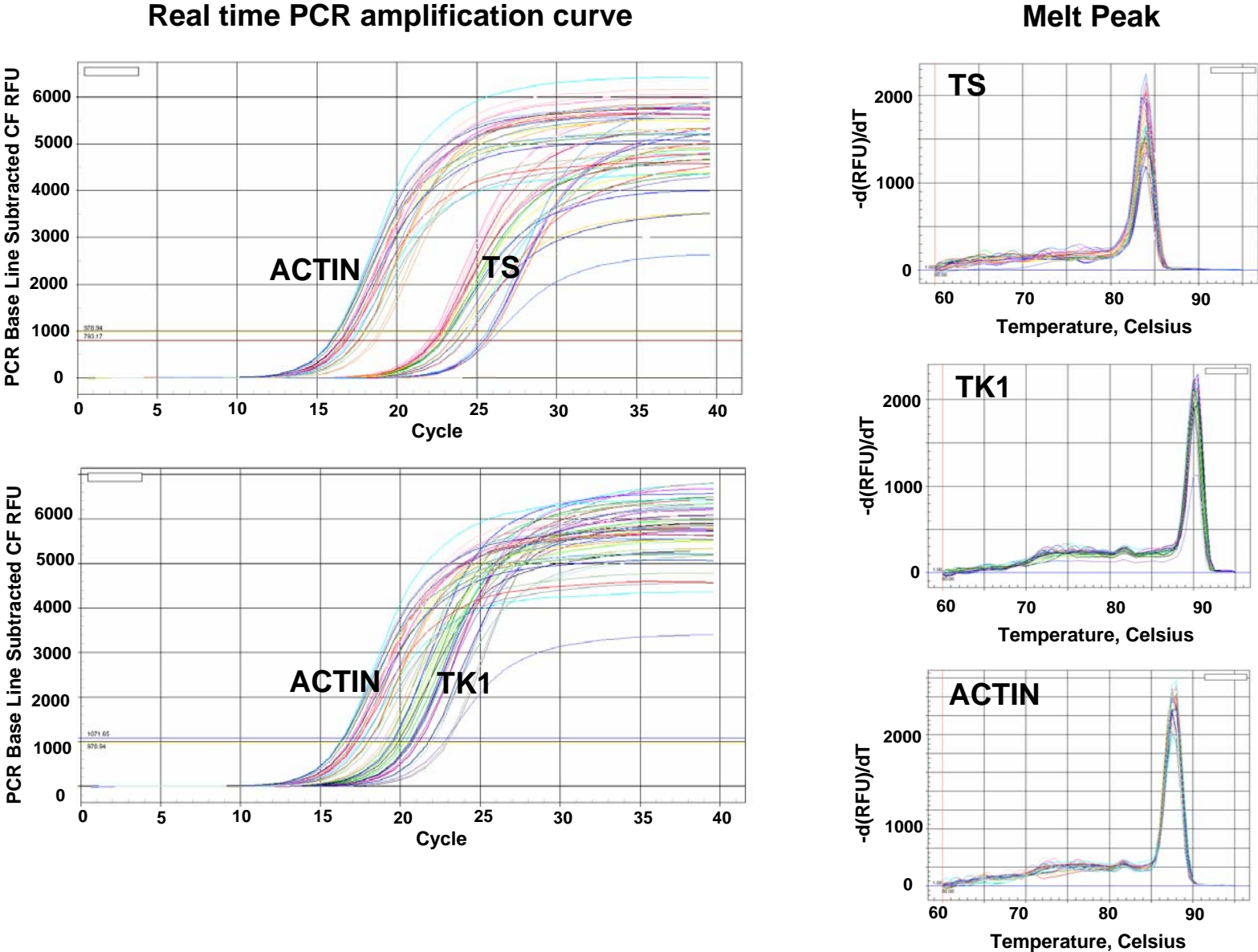

Real time PCR amplification curve

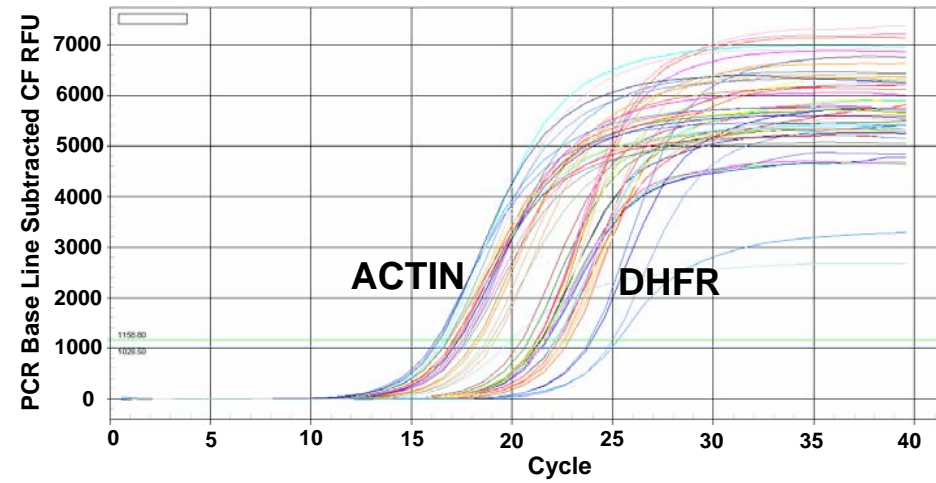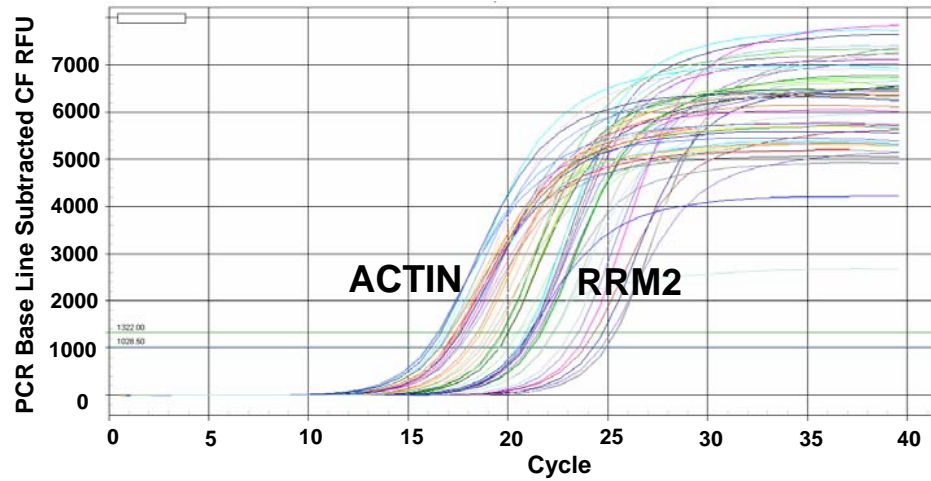

Melt Peak

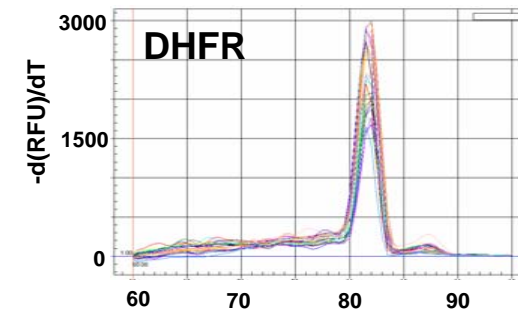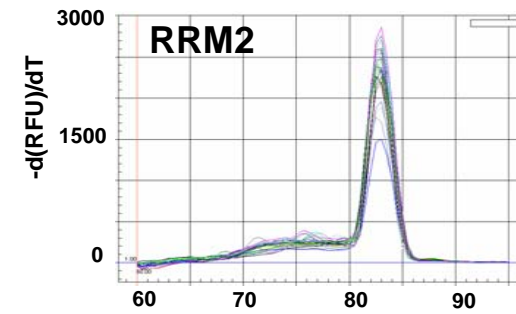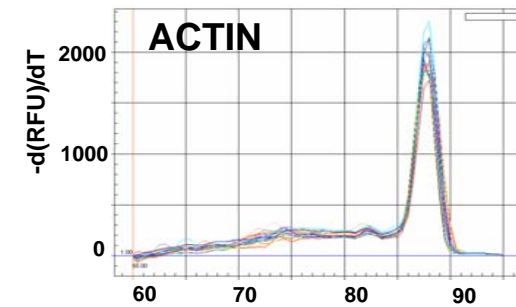

Supplement: Figure S1 — (0.22 MB PDF) [file pone.0005933.s001.pdf]
